# Machine Learning-Guided Development of Anti-Tuberculosis Dry Powder for Inhalation Prepared by Co-Spray Drying

**DOI:** 10.3390/pharmaceutics18020191

**Published:** 2026-02-01

**Authors:** Xiaoyun Hu, Xian Chen, Ziling Zhou, Aichao Wang, Xin Pan, Chuanbin Wu, Junhuang Jiang

**Affiliations:** 1 State Key Laboratory of Bioactive Molecules and Druggability Assessment, Guangdong Basic Research Center of Excellence for Natural Bioactive Molecules and Discovery of Innovative Drugs, College of Pharmacy, Jinan University, Guangzhou 511443, China; huxiaoyun2003@163.com (X.H.); chenxian@jnu.edu.cn (X.C.); zhouziling@stu2024.jnu.edu.cn (Z.Z.); 2 Tianjin Tianyao Pharmaceuticals Co., Ltd., Tianjin 300462, China; wac0785@126.com; 3 School of Pharmaceutical Sciences, Sun Yat-sen University, Guangzhou 510275, China; panxin2@mail.sysu.edu.cn

**Keywords:** tuberculosis, dry powder inhalation, spray drying, machine learning, aerodynamic performance

## Abstract

**Background/Objectives:** Tuberculosis (TB) remains a major global health threat. Current administration methods for anti-TB drugs, including oral or intravenous, suffer from systemic side effects, low lung distribution, and poor patient compliance. Dry powder inhalers (DPIs) offer a promising alternative. This study investigates the aerodynamic performance of co-spray-dried DPIs containing rifampin or pyrazinamide and amino acids by using machine learning. **Methods:** Firstly, 72 formulations were prepared by varying drug-amino acid combinations, molar ratios, and spray-drying parameters. Subsequently, the aerodynamic performance of all 72 formulations was evaluated using a Next Generation Impactor, and the solid-state characterizations of optimal DPIs were carried out. Finally, four machine learning (ML) models were successfully developed and were utilized to predict the fine particle dose (FPD), FPF, MMAD, and geometric standard deviation (GSD) of DPIs based on the high-quality in-house data above. **Results:** Key results showed that the aerodynamic performance of DPIs was highly dependent on the specific drug-amino acid combination, with rifampin-L-lysine acetate and pyrazinamide-L-leucine formulations achieving the highest fine particle fraction (FPF, 73.37%, 87.74%) and optimal mass median aerodynamic diameter (MMAD, 2.59 µm, 1.88 µm). Notably, XGBoost (v3.1.3) exhibited the best predictive performance, with R^2^ values ranging from 0.894 to 0.991 in the testing set for the four prediction tasks. Meanwhile, SHapley Additive exPlanations (v0.50.0) was used for model interpretability analysis. The molecular weights and LogP of the drug and amino acid were identified as two of the most important features affecting the prediction of FPD, FPF, MMAD, and GSD. **Conclusions**: This work demonstrates the feasibility of ML in accelerating the development of inhalable spray-dried anti-TB drugs by enabling the prediction of DPI formulations.

## 1. Introduction

Tuberculosis (TB) disease caused by the *M. tuberculosis* complex can affect human skin, pleura, meninges, lymph nodes, and any other organ, with the lung being the most commonly infected site [1,2]. According to the World Health Organization (WHO), TB ranks among the top 10 causes of death, and its annual incidence exceeds 10 million cases worldwide [3]. Moreover, TB disease (usually pulmonary TB) can be transmitted through aerosols generated when patients sneeze, cough, shout, or talk. The aerosolized droplet containing *M. tuberculosis* remains infectious even after prolonged suspension in the air over long distances [4]. Despite increased efforts in the fight to end TB in recent decades, this disease remains one of the major threats to global public health [2,4].

Compared with drug-resistant TB resistant to all available TB medicines, drug-susceptible TB can be treated with the WHO-recommended first-line regimens comprising isoniazid, rifampin, ethambutol, and pyrazinamide [2]. However, anti-TB drugs are primarily administered by intravenous injection or oral delivery [5,6]. Oral administration exhibits the first-pass effect, which results in decreased drug concentration among systematic or local action. Meanwhile, injection administration requires medical personnel and may cause discomfort to patients with poor compliance [5]. In addition, these conventional routes are both limited by significant systemic side effects. Based on the drug labels from the Food & Drug Administration (FDA), rifampin and pyrazinamide could result in gastrointestinal disturbances or hepatotoxicity after intravenous or oral administration [7,8,9]. Moreover, low drug lung deposition after oral or intravenous administration may rapidly result in drug resistance for pulmonary TB, further worsening the compliance for TB patients who need long-term drug therapy [5,10]. Therefore, it is necessary to develop a new route of drug administration.

To address the issues of traditional administration methods, there has been an increasing number of studies on the pulmonary drug delivery system (PDDS) used for treating TB. These studies also demonstrated the feasibility of pulmonary delivery for anti-TB [11,12,13]. Among the PDDS, dry powder inhalers (DPIs)—which deliver drugs in solid form—are inherently more stable than other inhalers during storage [14,15]. Furthermore, DPIs are both propellant-free and independent of hand-mouth coordination, thereby exhibiting great potential in the effective delivery of anti-TB drugs [16,17,18]. Nevertheless, particle properties, including aerodynamic diameter and morphology, significantly impact the aerodynamic performance and pulmonary deposition of DPIs. For optimal delivery efficiency, particles in DPIs should have an aerodynamic diameter between 1 and 5 μm. This size range allows particles to evade mucociliary clearance and deposit effectively in the airways [19,20,21,22,23]. Hence, it’s imperative to control particle properties precisely for DPIs. Co-spray drying is a bottom-up technique that is widely applied to prepare DPI particles with satisfactory properties by adjusting the formulations and process parameters [17,24,25]. During the spray drying process, amino acids, such as L-leucine, L-arginine, and trileucine, are typically utilized as co-formers, which act as aerodynamic performance enhancers [26,27,28,29,30,31,32,33,34]. Therefore, in this study, we propose to co-spray dry rifampin or pyrazinamide with a specific amino acid to prepare inhalable anti-TB drugs dry powder for inhalation.

In recent decades, Artificial Intelligence (AI) and Machine Learning (ML) have been extensively applied in pharmaceutical development and have revolutionized the paradigm of drug formulation development [35,36,37,38,39]. Compared to the traditional trial-and-error method, ML can significantly reduce time and cost with improved formulation development efficiency. It is reported that ML models show potential in the development of co-spray dried DPIs [18]. Specifically, Fröhlich et al. developed an ML model based on the molecular descriptors to predict dual-drug inhalable co-amorphous systems, with an accuracy of 0.79 [11]. Jiang et al. successfully employed various traditional ML and deep learning algorithms to predict the aerodynamic performance of DPIs prepared by thin-film freezing. The random forest and artificial neural network models achieved the best performance in predicting fine particle fraction (a mean absolute error of 7.251%) and mass median aerodynamic diameter (a mean absolute error of 0.393 µm), respectively [39]. Schmitt et al. used ensemble ML to predict the particle size distribution of spray-dried API particles, verifying the method’s feasibility with errors ranging from 7.7% to 18.6% [40]. Conventional ML modeling involves data collection, which typically employs literature mining through open access database. However, it faces challenges such as data accessibility and heterogeneity, which make it difficult to conduct targeted and in-depth research for the development of anti-TB drugs. This study adopts internal experimental results to establish a high-quality dataset, which can effectively guide the development of co-spray dried anti-TB DPIs.

In this study, we first obtained a high-quality dataset of anti-TB DPIs by conducting in-house experiments through spray drying. More importantly, we established ML models for predicting the aerodynamic performance of DPIs to accelerate the development of anti-TB DPIs. Concretely, rifampin and pyrazinamide are selected as two model drugs with different solubilities for ML model development. Rifampin is poorly water-soluble (1.4 mg/mL at 25 °C in water), and the latter is highly soluble (15 mg/mL at 25 °C in water) [41]. In addition, we chose L-leucine, L-arginine, and L-Lysine as excipients, which are often used in spray drying to enhance the aerodynamic performance of DPIs [26,27,28,29,30,31,32,33,34]. Considering that the molar ratio between the drug and the amino acid may reflect the essence of the possible interaction and eliminate the interference caused by molecular weight, three common molar ratios (2:1, 1:1, 1:2) were designed. Based on the process parameter range of the utilized spray-dryer and our prior experimental experience, the atomizer flow rate was set to 850, 800, 750, and 700 L/min. Following the principle of single-factor design (2 × 3 × 3 × 4), we prepared 72 DPI formulations by co-spray-drying rifampin or pyrazinamide with three amino acids at different molar ratios under four processing parameters. Subsequently, the aerodynamic performance of all DPI formulations was evaluated by Next Generation Impactor (NGI). Based on these experimental results, multiple ML models (e.g., random forests, extreme gradient boosting, support vector machine, and multilayer perceptron) were then established and evaluated to predict the aerodynamic performance of spray-dried DPIs.

## 2. Materials and Methods

### 2.1. Materials

**Figure 1:**
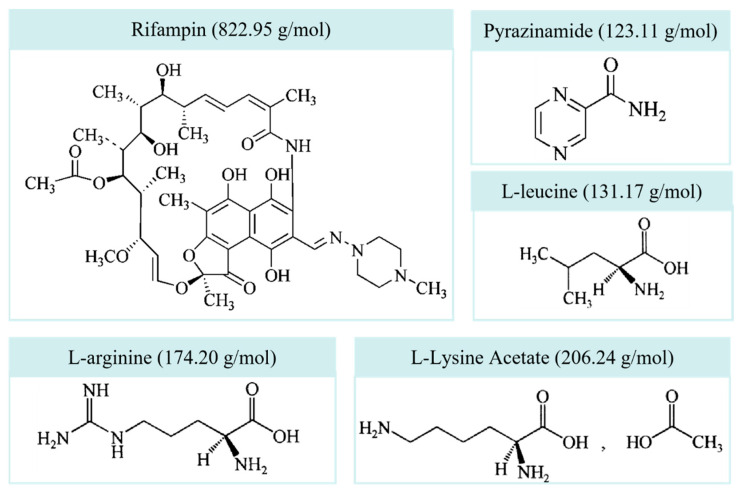
The structural formulas and molecular weights of model drugs and amino acids.

Rifampin (RIF, Figure 1) was purchased from Minzhong Pharmaceutical Co., Ltd. (Zhengzhou, China). Pyrazinamide (PYR) was purchased from Beijing Jingfeng Pharmaceutical (Shandong) Co., Ltd. (Zibo, China). L-arginine (LA), L-leucine (LL), and L-Lysine Acetate (LLA) were all products of Tianjin Tianyao Pharmaceuticals Co., Ltd. (Tianjin, China). Sodium thiosulfate [molecular weight (MW) 248.18 g/mol] was purchased from Hunan BKMAN Co., Ltd. (Changde, China). Potassium dihydrogen phosphate (MW 136.09 g/mol) was purchased from Tianjin Kermel Chemical Reagent Co., Ltd. (Tianjin, China). Tween 20 was from Aladdin Ltd. (Shanghai, China). Hydroxypropyl methylcellulose (HPMC) transparent size 3 capsules were from Qualicaps Co., Ltd. (Nara, Japan). All reagents were of reagent grade, except for ethanol (Tianjin Kermel Chemical Reagent Co., Ltd., Tianjin, China), methanol & acetonitrile (Tianjin Concord Technology Ltd., Tianjin, China), citric acid (Beijing MREDA Technology Co., Ltd., Beijing, China), and acetic acid (Anaqur Chemicals Supply Ltd., Wilmington, DE, USA), which were of high performance liquid chromatography (HPLC) grade or analytical grade. The unit-dose capsule-based DPI inhaler—Breezhaler^®^—was purchased from Novartis Pharma Stein AG (Risch, Switzerland) and was approved by the FDA for human use.

### 2.2. Spray Drying

RIF feed solutions were prepared by dissolving amino acids (LA, LL, or LLA) and sodium thiosulfate in nitrogen-filling-treated 60% (*v*/*v*) ethanol aqueous solution and then dissolving rifampin into the solution above. Sodium thiosulfate was added as a solubility enhancer at a concentration of 2.0% of RIF. For PYR feed solutions, amino acids (LA, LL, or LLA) and pyrazinamide were added and dissolved sequentially in 50% (*v*/*v*) ethanol aqueous solution. The molar ratio of drug to amino acid was kept at 2:1, 1:1, or 1:2, and the solid content of all the solutions was fixed at 1% (*m*/*v*).

All feed solutions were added into the YM-6000Y spray-dryer (Shanghai YuMing Instrument Co., Ltd., Shanghai, China) at the rate of 4 mL/min. The inlet air temperature was 110 °C, and the atomizer was set as 850 L/min, 800 L/min, 750 L/min, and 700 L/min. A total of 72 DPI formulations were prepared.

### 2.3. In Vitro Aerodynamic Performance

Next Generation Impactor (NGI) from Copley (Nottingham, UK) and Breezhaler^®^ were used to determine the in vitro aerodynamic performance of the spray-dried powders. To minimize particle bouncing, all eight collection plates were coated with 8% (*v*/*v*) Tween 20 in methanol and allowed to dry before the operation. A capsule containing 5.50 mg (for RIF DPIs) or 3.85 mg (for PYR DPIs) API was loaded into the inhaler and dispersed at a 4 kPa pressure drop and a 90 L/min air flow rate. Two capsules were fabricated for the RIF formulation, and one capsule was made for the PYR formulation. 50% (*v*/*v*) methanol aqueous solution (for RIF DPIs) or ultrapure water (for PYR DPIs) was used to recover the deposited powder on the capsule, inhaler, adapter, throat, pre-separator (PS), stage 1 (S1)–stage 7 (S7), and micro-orifice collector (MOC). The quantitative analysis of RIF or PYR was performed by HPLC, as specified in Table S1. Each DPI was conducted in triplicate, and the results are presented as the mean ± standard deviation.

Fine particle dose (FPD) is defined as the mass of the drug particles having an aerodynamic diameter smaller than 5 μm (calculated by interpolation of the NGI data graph (cumulative mass vs. D50)). Fine particle fraction (FPF) refers to the percentage of FPD relative to the emitted dose (i.e., drugs recovered from the adapter to MOC). Emitted fraction (EF) is the percentage of the dose released from the inhaler to the total recovered dose. The mass median aerodynamic diameter (MMAD) represents the aerodynamic diameter at which the cumulative mass distribution curve reaches 50%. Geometric standard deviation (GSD) means the spread of an aerodynamic particle size distribution. The FPD, FPF, MMAD, and GSD in this experiment were calculated using CITDAS^®^ software (version 3.10, Copley Scientific Ltd., Nottingham, UK).

### 2.4. Physicochemical Characterization

#### 2.4.1. X-Ray Diffraction (XRD)

XRD was performed using Rigaku UIV from Japan, with a Cu Kα X-ray source at 40 kV and 40 mA. The PXRD spectra of the samples were recorded over 5–60° with a step size of 0.02° and a rate of 8°/min.

#### 2.4.2. Differential Scanning Calorimetry (DSC)

The thermal behavior of DPIs was further detected by TA Discovery DSC 25 (TA Instruments Products and Solutions, New Castle, DE, USA). About 2–4 mg of the sample was loaded into an aluminum T-zero pan and sealed with a lid. The pans were heated from 40 °C to 330 °C at a rate of 10 °C/min under a 50 mL/min nitrogen flow.

#### 2.4.3. Scanning Electron Microscopy (SEM)

The powder’s surface morphology was determined by SEM Zeiss MERLIN Compact from Germany at 5 kV and 5.00 KX & 2.00 KX magnifications. Each DPI powder was mounted on carbon tape and subsequently sputter-coated with gold for about 70–80 s before imaging.

### 2.5. Machine Learning Model Development

#### 2.5.1. Data Preparation

We employed the internal dataset of 72 DPI formulations for ML modeling. Table 1 details the features used in the predictive model for each formulation, including 16 feature variables and 4 target variables. Feature variables include key physicochemical properties of drug molecules and amino acids, including molecular weight (MolWt), logarithm of the Partition Coefficient (MolLogP), topological polar surface area (TPSA), as well as formulation process parameters such as ratio of drug-amino acid and atomization gas flow rate. The target variables are FPD, FPF, MMAD, and GSD.

Additionally, data preprocessing employs standardization methods to eliminate the impact of differences in feature scales on model training. The equation is as follows:

(1) $$x^{\prime }=\frac{x-\mu }{\sigma }$$

where x is the raw feature value, μ is the mean of the feature, σ is the standard deviation of the feature, and $x^{\prime }$ is the standardized feature value.

Ultimately, the preprocessed dataset was divided into a training subset (70%) and a testing subset (30%). Because the dataset is relatively small, we employed 5-fold cross-validation to model and optimize the model’s hyperparameters in order to comprehensively evaluate its generalization capability. The training set was randomly divided into five parts. Four parts are used as the training set in turn, while one part is used as the validation set. The final result is the average of the five validation runs.

#### 2.5.2. Model Construction

This study employed four ML algorithms to construct regression models: random forests (RF), extreme gradient boosting (XGBoost), support vector machine (SVM), and multilayer perceptron (MLP). To predict four target variables, MultiOutputRegressor was employed to train a separate machine learning model for each target variable. However, these models shared a common set of hyperparameters to maintain consistency in model architecture. Additionally, an MLP model was constructed with two hidden layers (containing 20 and 10 neurons, respectively), using the tanh activation function and stochastic gradient descent (SGD) as the optimizer for training. We employed random search to fine-tune these models by adjusting the hyperparameters of different algorithms. The hyperparameters of models are shown in Table 2. All models were developed using Python (v3.12.12) and implemented with Scikit-learn (v1.6.1) and related libraries.

#### 2.5.3. Evaluation Criteria and Interpretability

In machine learning regression tasks, commonly used statistical metrics for evaluating model prediction performance include the coefficient of determination (R^2^), mean absolute error (MAE), and root mean square error (RMSE). To comprehensively evaluate the accuracy and reliability of the model’s performance, this study employed these statistical metrics for comprehensive quantitative analysis, as shown in Equations (2)–(4).

(2) $$R^{2}=1-\frac{\sum_{i=1}^{n}{\left(y_{i}-{\hat{y}}_{i}\right)}^{2}}{\sum_{i=1}^{n}{\left(y_{i}-\bar{y}\right)}^{2}}$$

(3) $$MAE=\frac{1}{n}\overset{n}{\underset{i=1}{\sum }}\left|{\hat{y}}_{l}-y_{i}\right|$$

(4) $$RMSE=\sqrt{\frac{1}{n}\sum_{i=1}^{n}{\left(y_{i}-{\hat{y}}_{i}\right)}^{2}}$$

where $y_{i}$ is the actual value, ${\hat{y}}_{i}$ is the predicted value, $\bar{y}$ is the mean of actual values, and n is the total number of samples.

Additionally, we employed SHAP (SHapley Additive exPlanations) for model interpretability analysis in this study [42,43]. SHAP employs game theory techniques to compute a SHAP value for each feature, quantifying the marginal contribution of that feature to a single prediction outcome. Through local and global interpretability analysis, gain deep insights into the model’s decision-making mechanisms and the contribution of different features to prediction outcomes.

## 3. Results

### 3.1. Aerodynamic Performance

**Figure 2:**
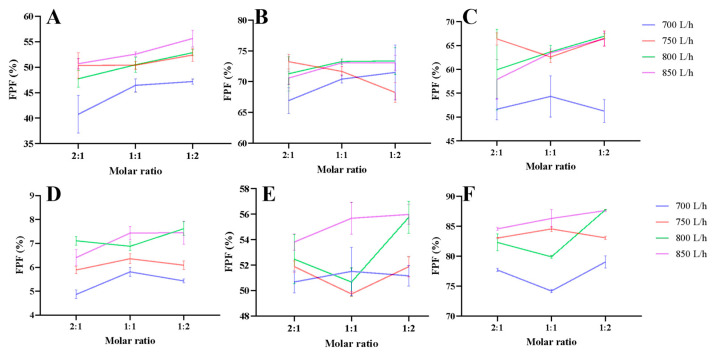
FPF of spray-dried RIF-LA (**A**), RIF-LLA (**B**), RIF-LL (**C**), PYR-LA (**D**), PYR-LLA (**E**), PYR-LL (**F**) DPIs (RIF: rifampin, PYR: pyrazinamide, LA: L-arginine, LLA: L-Lysine Acetate, LL: L-leucine).

**Figure 3:**
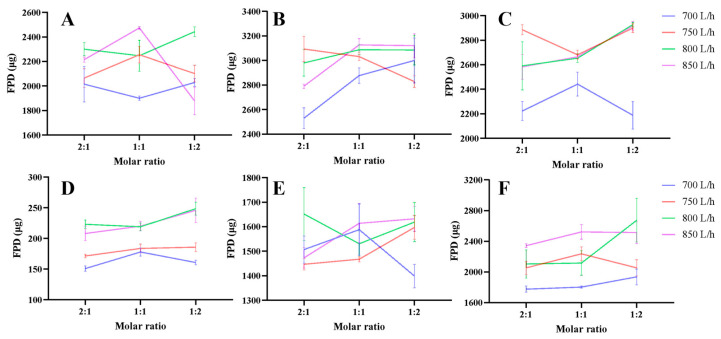
FPD of spray-dried RIF-LA (**A**), RIF-LLA (**B**), RIF-LL (**C**), PYR-LA (**D**), PYR-LLA (**E**), PYR-LL (**F**) DPIs.

The aerodynamic performance results of RIF DPIs were summarized in Table S2 and Figure 2 and Figure 3. For all 36 formulations of RIF DPIs, the FPD, FPF, MMAD, and GSD were 1878.80–3127.32 µg, 40.77–73.37%, 2.59–3.22 µm, and 1.59–1.79, respectively. The EF of 36 formulations was higher than 81.00% (Table S2). Among the combinations of rifampicin with three amino acids, RIF-LLA DPIs displayed the best aerodynamic behavior with the highest FPD (2530.53–3127.32 µg), the highest FPF (66.92–73.37%), and the smallest MMAD (2.59–2.64 µm). RIF-LA DPIs showed the worst aerodynamic behavior with the lowest FPD (1878.80–2473.71 µg), the lowest FPF (40.77–55.65%), and the largest MMAD (2.71–3.22 µm) (Table S2). The RIF-LL aerodynamic performance was at a medium level. For RIF LA and RIF-LL DPIs, elevating the atomizing air flow rate from 700 L/h to 750 L/h might increase the FPF to varying degrees significantly (3.96–15.23%). At the same time, the FPF couldn’t obviously improve even though the atomizing air flow rate boosted from 750 L/h to 850 L/h (an increase of less than 3%) (Figure 2A,C). The influence of atomizing air flow rates on the FPF varied with the ratio of RIF to amino acid for the RIF-LLA (Figure 2B). Under different atomizing air flow rates, the FPF of RIF-LA DPIs prepared with a molar ratio of RIF to LA of 1:2, 1:1, and 2:1 decreased successively (Figure 2A). However, this phenomenon was only observed at some of the atomizing air flow rates for the RIF-LLA and RIF-LL formulations (Figure 2B,C).

The aerodynamic performance results of PYR DPIs were summarized in Table S3 and Figure 2 and Figure 3. For all 36 formulations of PYR DPIs, the FPD, FPF, MMAD, and GSD were 150.88–2674.31 µg, 4.87–87.74%, 1.88–6.45 µm, and 1.57–2.13, respectively. The EF of 36 formulations was higher than 79.00% (Table S3). Among the combinations of pyrazinamide with three amino acids, PYR-LL DPIs displayed the best aerodynamic behavior with the highest FPD (1777.31–2674.31 µg), the highest FPF (74.20–87.74%), and the smallest MMAD (1.88–1.90 µm). PYR-LA DPIs displayed the worst aerodynamic behavior with the lowest FPD (150.88–248.61 µg), the lowest FPF (4.87–7.62%), and the largest MMAD (4.97–6.45 µm) (Table S3). The PYR-LLA aerodynamic performance was at a medium level. For PYR-LL DPIs, elevating the atomizing air flow rate from 700 L/h to 750 L/h increased the FPF to varying degrees (4.02–10.36%). When the atomizing air flow rate was higher than 750 L/h, the variation in FPF showed no discernible trend (Figure 2F). For the PYR-LLA formulation, only when the atomizing air flow rate increased to 850 L/h, the FPF was improved obviously (Figure 2E). Moreover, the atomizing air flow rate had a very minor impact on the FPF of PYR-LA DPIs (Figure 2D). The impact of molar ratios of PYR to amino acids on the FPF did not exhibit a discernible pattern (Figure 2D–F).

### 3.2. Characterization of DPIs

The results of NGI demonstrated that RIF-LLA and PYR-LL DPIs at 850 L/h had the best aerodynamic performance with the highest FPF and the lowest MMAD (Tables S2 and S3 and Figure 2 and Figure 3). Therefore, we examined these optimal formulations with XRD, DSC, and SEM.

**Figure 4:**
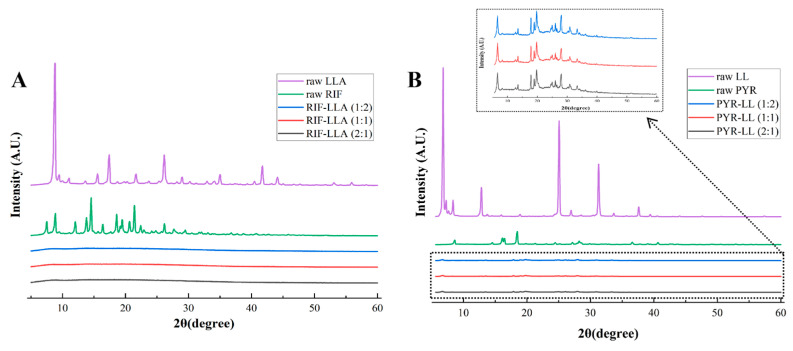
(**A**) XRD patterns of raw LLA, raw RIF, RIF-LLA 1:2, RIF-LLA 1:1, and RIF-LLA 2:1 (from top to bottom). (**B**) XRD patterns of raw LL, raw PYR, PYR-LL 1:2, PYR-LL 1:1, and PYR-LL 2:1 (from top to bottom).

As shown in Figure 4, raw RIF, PYR, LLA, and LL existed as crystalline materials. Concretely, raw RIF showed peaks at 8.86°, 13.82°, 14.54°, 18.60°, and 21.42°; raw PYR at 8.00°, 15.50°, 15.82°, 17.84°, and 27.60°; raw LLA at 8.80°, 17.42°, and 26.14°; and raw LL at 6.18°, 12.20°, 24.44°, and 30.66°. Interestingly, all co-spray-dried RIF-LLA DPIs with various ratios (2:1, 1:1, 1:2) converted to an amorphous state, exhibiting no Bragg peaks between 5–60° (Figure 4A). Nevertheless, all co-spray-dried PYR-LL DPIs at all ratios (2:1, 1:1, 1:2) remained crystalline, having Bragg peaks at 6.08°, 19.14°, 24.38°, 27.44°, and 30.64° (Figure 4B).

**Figure 5:**
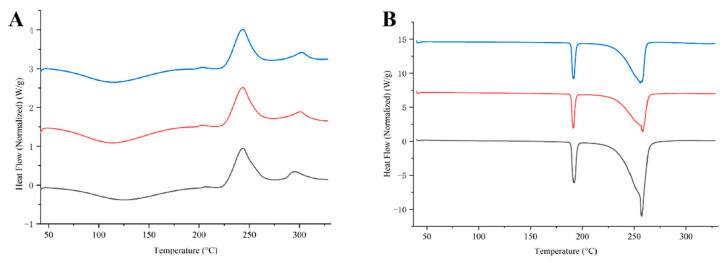
(**A**) DSC heat flow curves of RIF-LLA 1:2, RIF-LLA 1:1, and RIF-LLA 2:1 (from top to bottom). (**B**) DSC heat flow curves of PYR-LL 1:2, PYR-LL 1:1, PYR-LL 2:1 (from top to bottom).

DSC thermograms of RIF and PYR DPIs are shown in Figure 5. For all RIF-LLA DPIs, a broad endothermic peak (peak approximately 120 °C) caused by the evaporation of solvents was observed. This peak was followed by an exothermic peak attributed to recrystallization (peak around 243 °C) (Figure 5A). By contrast, all PYR-LL DPIs exhibited two sharp endothermic peaks, one melting peak at about 191 °C and one degradation peak at about 256 °C (Figure 5B). For the DPIs containing the same API, the comparable thermal behaviors at different ratios (2:1, 1:1, 1:2) indicated their similar solid-state characteristics. However, there was a significant difference between RIF DPIs and PYR DPIs.

**Figure 6:**
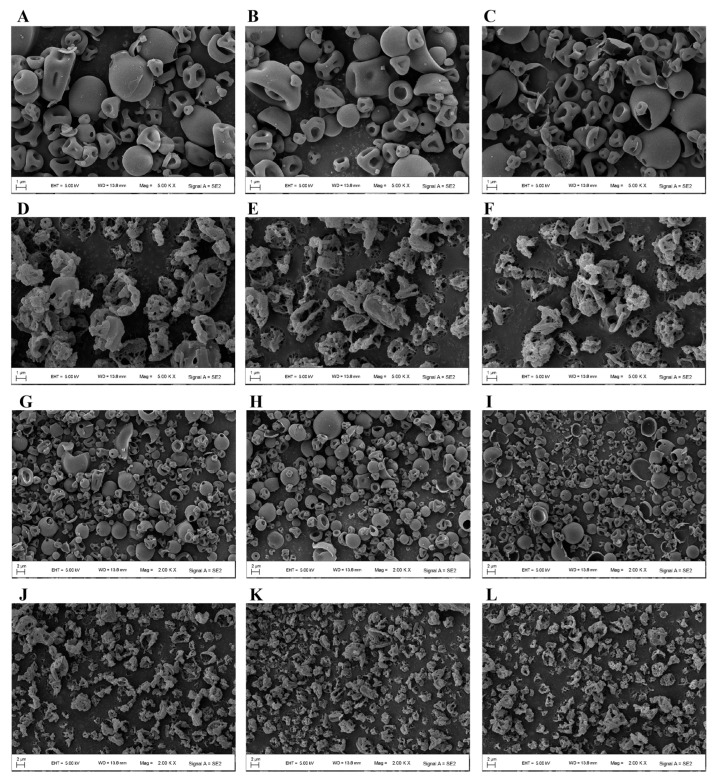
SEM images (Magnification factors 5.00 KX and 2.00 KX) of RIF-LLA 2:1 (**A**,**G**), RIF-LLA 1:1 (**B**,**H**), RIF-LLA 1:2 (**C**,**I**), PYR-LL 2:1 (**D**,**J**), PYR-LL 1:1 (**E**,**K**), and PYR-LL 1:2 (**F**,**L**).

For all RIF-LLA DPIs, particles were mostly irregular and corrugated or invaginated, along with a small number of particles that were smooth and near spherical. The higher the content of LLA, the more irregular and invaginated particles there were (Figure 6A–C,G–I). However, the particles in the PYR-LL formulation presented fragmented, broken, or layered structures. An increase in LL led to an increase in debris (Figure 6D–F,J–L).

### 3.3. Model Performance

We successfully applied multiple ML models to predict the aerodynamic performance of spray-dried DPI. For the training set, five-fold cross-validation was employed for modeling and optimization, while the testing set was used to evaluate model performance. We calculated various evaluation metrics, including R^2^, MAE, and RMSE, to characterize the predictive performance of each model. The predictive performance of all models is shown in Table 3. All models demonstrated satisfactory performance across the training, cross-validation, and testing datasets, achieving average R^2^ values exceeding 0.88 for the four target variables (FPD, FPF, MMAD, GSD). This indicated robust fitting capability and excellent generalization ability. Specifically, for FPD prediction, the performance of the four models is relatively similar. For the prediction of FPF and MMAD, XGBoost exhibited the best performance, achieving a slightly higher R^2^ among the four ML models and showing a smaller deviation between predicted values and actual values. For GSD prediction, SVM performed relatively worse on the testing set. (R^2^: 0.881; MAE: 0.041; RMSE: 0.052). Overall, XGBoost achieved the optimal and robust predictive performance, producing the lowest overall errors. Therefore, we selected XGBoost as the optimal model for further analysis.

### 3.4. Model Interpretation

**Figure 7:**
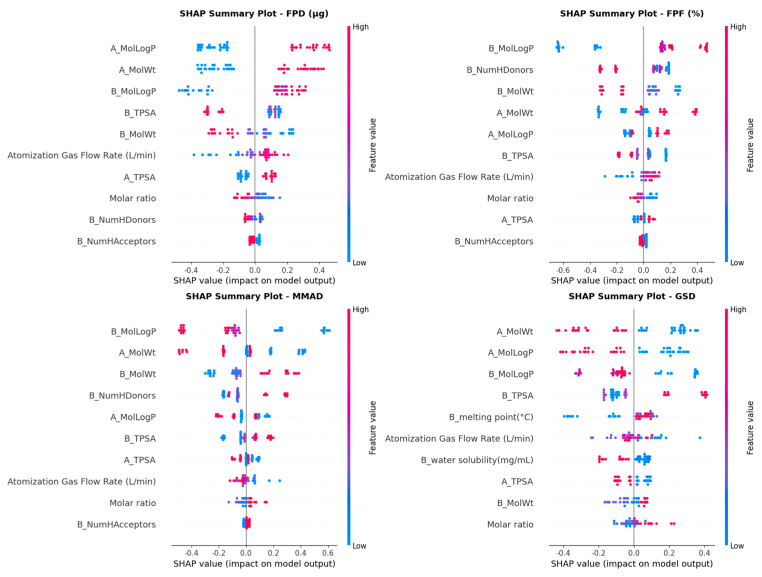
SHAP summary plots for the XGBoost model predictions. The prefix “A_” indicates drug-related parameters, while “B_” indicates amino acid-related parameters.

To systematically evaluate the influence of each feature on aerodynamic performance, the SHAP method was employed to analyze the interpretability of the four target variables (FPD, FPF, MMAD, GSD). As shown in Figure 7, four SHAP summary plots were generated by calculating SHAP values for each target variable. Through analysis combined with the feature importance heatmap (Figure S1), it was found that the LogP of amino acid (B_MolLogP) is the most significant feature, showing the strongest contribution on FPF and MMAD, while also exhibiting considerable impact on FPD and GSD. Specifically, FPD is primarily influenced by the drug’s LogP and molecular weight (A_MolLogP and A_MolWt), which both exhibited positive correlations. Both the number of hydrogen bond donors and the molecular weight of amino acids showed significant negative effects on the prediction of FPF. MMAD and GSD were closely related to the molecular weight and LogP of drugs and amino acids. The melting point of amino acids had a slightly higher impact on GSD prediction but had almost no effect on other targets. Furthermore, it was found that the atomization gas flow rate exhibited a positive correlation with the predicted values of FPD and FPF, while showing a negative correlation with the predicted values of MMAD and GSD. However, the molar ratio of drug to amino acid exhibited the opposite trend in its influence on the four target variables. Overall, among all feature variables investigated in this study, the molecular weights and LogP of drugs and amino acids exhibited the most significant impact on aerodynamic properties, ranking among the top five for almost all four prediction targets.

**Figure 8:**
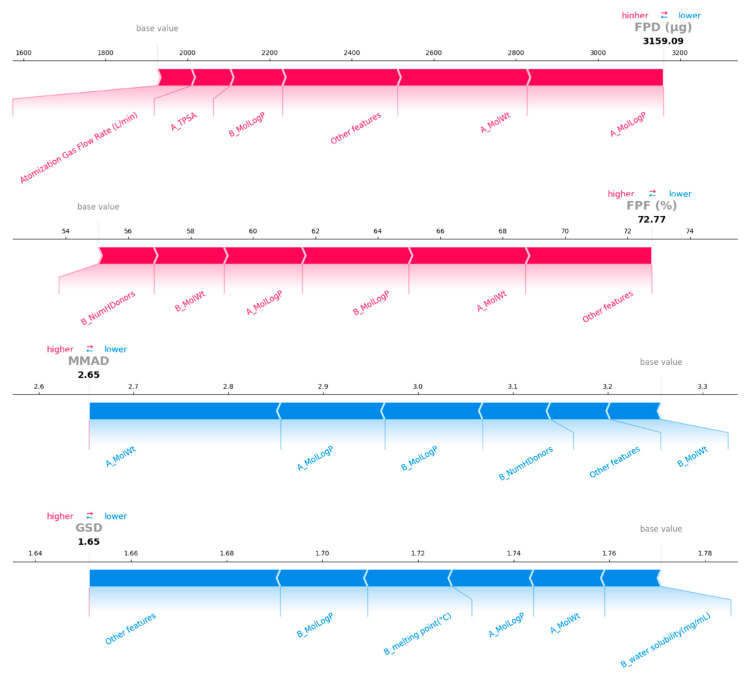
SHAP force plots for the RIF-LLA formulation. The prefix “A_” indicates drug-related parameters, while “B_” indicates amino acid-related parameters.

**Figure 9:**
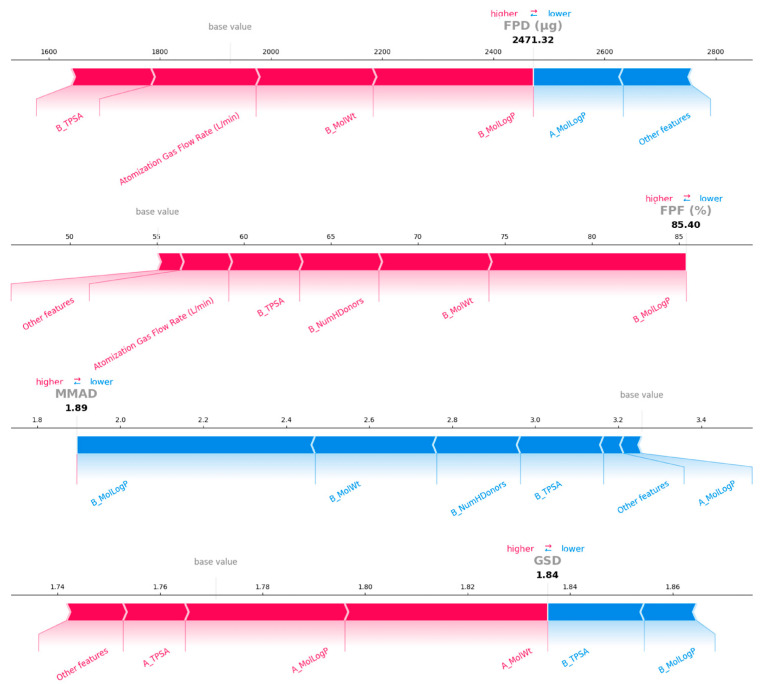
SHAP force plots for the PYR-LL formulation. The prefix “A_” indicates drug-related parameters, while “B_” indicates amino acid-related parameters.

Furthermore, this study employed local interpretability methods to further analyze the optimal formulations for rifampicin and pyrazinamide (Table 4), exploring the predictive mechanisms for the aerodynamic performance of inhalation particles of specific drugs. Figure 8 and Figure 9 displayed the top five most important features for each target variable, along with the positive (red) and negative (blue) contributions of each feature to the prediction results for specific formulations. Other features represent the sum of contribution values for all remaining features not displayed. The length of the band represents the degree to which each feature contributes to the model output.

For the RIF-LLA formulation, the results indicated that the top five most significant features primarily include the molecular weight, LogP, and TPSA of the drug and amino acid. Specifically, these features exhibited a positive influence on the prediction of FPD and FFP, while having a negative influence on the prediction of MMAD and GSD. We observe that when the value of A_MolWt was large in the global SHAP analysis, it tended to make a positive contribution to the prediction, increasing the FPD predicted value. The drug (rifampin, MW 822.95 g/mol in this formulation has a relatively high molecular weight. As shown in Figure 8, it made a positive contribution to the prediction of FPD, consistent with the conclusions of the global interpretability analysis. Moreover, the process parameter—atomization gas flow rate—exhibited a positive contribution to FPD prediction, suggesting that appropriately increasing gas flow can enhance the FPD of RIF-LLA formulation. In summary, interpretability analysis indicated that the top five most important features across the four target variables (FPD, FPF, MMAD, GSD) were largely consistent, primarily including molecular weight, LogP, and TPSA of drugs and amino acids. However, the direction of these features’ contributions to different target variables is not consistent. Thus, it is evident that while key physicochemical characteristics exhibit certain commonalities in influencing different aerosol performance metrics, their specific contribution direction remains dependent on the target variable being predicted.

For the PYR-LL formulation, beyond the previously mentioned features, the number of hydrogen bond donors of amino acid(B_NumHDonors) also exhibited a medium-strength influence (0.1 < |SHAP| < 0.2) on the prediction of aerodynamic performance, as shown in Figure 9. The LogP and molecular weight of amino acids are the top two features for the prediction of FPD and FPF. In contrast, the amino acid’s molecular weight and LogP exhibited a significant negative effect on the prediction of MMAD. Similarly, the drug in this formulation is pyrazinamide, with a LogP of −0.4245, a relatively low value within the entire dataset. In the FPD prediction, this feature made a negative contribution to the prediction, consistent with the results of the previous analysis. This further validated that the model maintained good consistency between local and global interpretation. In addition, the analysis results also showed that appropriately increasing the atomization gas flow rate helps to enhance both FPD and FPF concurrently, suggesting that this process parameter played a positive role in optimizing the atomization performance of this formulation.

## 4. Discussion

### 4.1. Inhalable Property of Spray-Dried DPIs

Rifampicin and pyrazinamide, as first-line anti-TB drugs, are selected as model drugs to prepare DPI formulations. We systematically study the aerodynamic performance of spray-dried DPIs containing RIF or PYR with three amino acids (LA, LL, LLA) at three ratios and four atomizing air flow rates. The results show that most of the powder can be released from the inhaler for all 72 formulations. The aerodynamic performance is closely related to the drug–amino acid combination: RIF-LLA DPIs exhibit the highest FPF (66.92–73.37%) and the smallest MMAD (2.59–2.64 µm), while PYR-LL DPIs achieve the highest FPF (74.20–87.74%) and the lowest MMAD (1.88–1.90 µm). We speculate that this is related to the respective physicochemical properties of the drugs and amino acids. In addition, increasing the atomizing air flow rate from 700 L/h to 750 L/h improves the FPF, but further increases beyond 750 L/h show negligible impact, indicating an optimal processing window.

Further characterization of the optimal formulations (RIF-LLA and PYR-LL) by XRD, DSC, and SEM reveals marked differences in their solid-state properties. All RIF-LLA formulations are amorphous based on XRD patterns, further confirmed by their DSC thermograms, in which there is no sharp endothermic peak. On the contrary, the PYR-LL formulations remain crystalline. There are Bragg peaks in the XRD spectrum of PYR-LL DPIs close to those of raw crystalline pyrazinamide and L-leucine. Moreover, the melting endothermic peak at around 191 °C in the PYR-LL DPIs is comparable to the previously reported melting point of pyrazinamide (188.5–188.8 °C) [44]. These findings indicate that both pyrazinamide and L-leucine may have partially crystallized after co-spray drying. For amorphous RIF DPIs, factors such as moisture and temperature may cause recrystallization of API, further influencing the quality and therapeutic effects of DPIs. Therefore, it is necessary to focus on their stability in the future.

SEM images showed that RIF-LLA particles were mostly irregular and corrugated, with more invaginated structures at higher LLA content; PYR-LL particles appeared fragmented or layered, with increased debris at higher LL content. This difference may be attributed to the variations in the solubility and molecular weight of these materials. During the spray drying, the poorly water-soluble component with large molecular weight tends to diffuse, aggregate, and solidify on the surface of the droplets. Meanwhile, the soluble components with small molecular weight are inclined to migrate and concentrate into the interior of the droplet [17,24]. For RIF-LLA DPIs, the molecular weight of poorly water-soluble RIF (822.95 g/mol) is around 4 times that of soluble LLA (206.24 g/mol). However, the molecular weight of poorly water-soluble LL (131.17 g/mol) is very close to that of soluble PYR (123.11 g/mol) in PYR-LL DPIs. Under the same drying process, the particles in the RIF-LLA formulations are more capable of forming a thick and solid shell compared to those in the PYR-LL formulations, and they are less likely to collapse or break.

### 4.2. Model Development and Selection

In this study, we proposed interpretable ML models to predict the aerodynamic performance of spray-dried DPIs. In the four prediction tasks (FPD, FPF, MMAD, GSD), XGBoost demonstrated the best performance on the test subset. Overall, compared to other models, this model achieved robust performance and good generalization capability. Therefore, this model was employed for further analysis. XGBoost is a gradient boosting model based on decision tree algorithms. It employs a regularized objective function to effectively prevent overfitting and reduce variance by aggregating gradient-boosted weights from multiple decision trees. Thus, it can handle nonlinearity and exhibits strong robustness [45]. Furthermore, we observed relatively high MAE and RMSE values for FPD, primarily due to its wide numerical range (150.88–3127.32 μg). Despite the large absolute error values, the R^2^ value exceeding 0.9 indicated excellent predictive accuracy when considering the scale of FPD.

We conducted further interpretability analysis on the best model (XGBoost). Through local and global interpretability analysis, we identified key features influencing aerodynamic performance (Figure 7, Figure 8 and Figure 9). SHAP analysis not only quantified the importance of each feature but also revealed the key factors influencing the aerodynamic performance of inhalation formulations and their direction of effect. Analysis results indicated that the molecular weights of the drug and amino acids have a significant impact on aerodynamic performance. During co-spray drying, the competition between the evaporation rate of liquid droplets and the diffusion rate of solutes determines the final structure of the particles [46]. Molecular weight influences particle structure by affecting diffusion rates. The diffusion coefficient of high-molecular-weight components is relatively low, leading to surface enrichment and the formation of hollow particles. This tends to result in low-density, invaginated spherical structures. In contrast, low-molecular-weight components diffuse more rapidly and tend to form dense particles.

For the RIF-LLA formulation, the molecular weight difference between the drug and amino acid is significant, with rifampicin being approximately four times heavier than lysine. As shown in SEM images (Figure 6), RIF-LL particles exhibited an invaginated hollow structure, indicating that rifampicin’s molecular weight dominated the particles’ low-density characteristics. This led to a smaller aerodynamic particle size, enabling efficient delivery to the lungs. This result is consistent with the outcomes shown in Figure 8, where the molecular weight of the drug (rifampicin) contributed positively to both FPD and FPF predictions. For the PYR-LL formulation, the MolLogP, NumHDonors, and TPSA values of the amino acid (leucine) all made positive contributions to the FPF prediction (Figure 9). These three features are all closely related to the hydrophobicity of amino acids. Previous studies have reported that leucine exhibits hydrophobicity and surface activity, leading to its surface enrichment on particles during co-spray drying [14]. Our experimental results also demonstrated that the PYR-LL particles exhibited a certain surface roughness, which reduces the contact area between particles and significantly lowers van der Waals and electrostatic forces. Ultimately, it enhances the efficiency of drug delivery to the lungs.

In summary, the ML model developed in our study has the following advantages: First, the model is trained entirely on high-quality data generated from in-house experiments, ensuring both the reliability and relevance of the data. Second, no publicly available research has reported predictive models for anti-tuberculosis DPI formulation prepared by spray drying, thus making this study the first exploration in this field. More importantly, the developed models demonstrated robust predictive capabilities with high accuracy. The best model (XGBoost) achieved an R^2^ value above 0.89 across all four prediction tasks. Furthermore, this study provides supporting evidence for screening and decision-making regarding DPI formulations based on model interpretability analysis, thereby enhancing the model’s practical guidance value. However, this study has certain limitations. First, the sample size is limited (*n* = 72), and the dataset only includes two model drugs and three amino acids, resulting in a narrow chemical space covered by the model. Therefore, the predictive capabilities of the current model may be difficult to fully generalize to broader API systems, with its generalizability constrained by data limitations. In further research, it is possible to expand the range of chemical space by integrating larger datasets, thereby enhancing the ML model’s generalization ability and prediction reliability.

## 5. Conclusions

This study aims to investigate and predict the aerodynamic performance of spray-dried anti-TB DPIs employing machine learning. We prepared 72 formulations containing a first-line anti-TB drug (rifampicin or pyrazinamide) and an amino acid (LA, LL, LLA) at three ratios and four atomizing air flow rates. Key findings revealed that aerodynamic performance depended strongly on the drug-amino acid combination: RIF-LLA and PYR-LL DPIs achieved the highest FPF (up to 73.37%, 87.74%) and smallest MMAD (as low as 2.59 µm, 1.88 µm) among all formulations. The influence of the ratio of drug to amino acid and atomizing air flow rates was complex. Solid-state characterization showed that RIF-LLA formulations were amorphous with irregular, corrugated particles, while PYR-LL formulations remained crystalline with fragmented morphology. These structural differences were attributed to the molecular weight and solubility disparities of the components, which in turn influenced shell formation during spray drying. It is especially worth noting that we have established a predictive model for the aerodynamic performance of inhaled particles based entirely on in-house high-quality data. Among all models, the XGBoost model achieved an R^2^ value above 0.89 on the test set, exhibiting excellent predictive accuracy and strong generalization capabilities. Unlike models relying on public datasets, all data in this study were generated under strictly controlled experimental conditions, ensuring consistency in experimental settings and minimizing error. In the future, we can enhance model robustness by expanding the dataset scale and exploring the application of advanced algorithms such as deep learning. Our study verified the potential of ML to predict the aerodynamic performance of co-spray dried anti-TB DPIs, which will significantly accelerate the development of the pulmonary delivery of anti-TB drugs.

## Figures and Tables

**Table 1:** Features and target variables applied in predictive ML models (NumHDonors, Number of Hydrogen Bond Donors; NumHAcceptors, Number of Hydrogen Bond Acceptors).

Categories	Features
Drug	MolWt, MolLogP, TPSA, NumHDonors, NumHAcceptors, melting point (°C), water solubility (mg/mL)
Amino acid	MolWt, MolLogP, TPSA, NumHDonors, NumHAcceptors, melting point (°C), water solubility (mg/mL)
Conditions	Molar ratio, Atomization Gas Flow Rate (L/min)
Target variables	FPD (μg), FPF (%), MMAD (µm), GSD

**Table 2:** Features and target variables applied in predictive ML models.

ML Algorithms	Hyperparameters					
RF	n_estimators	max_depth	min_samples_leaf	min_samples_split	max_samples	
	150	10	2	4	0.9	
XGBoost	learning_rate	n_estimators	max_depth	subsample	colsample_bytree	min_child_weight
	0.2	100	3	0.9	0.8	1
SVM	Kernel function	C	gamma			
	rbf	1	auto			
MLP	hidden_layer_sizes	activation	optimizer	learning_rate		
	(20, 10)	tanh	SGD	0.1		

**Table 3:** ML model performance for predicting FPD, FPF, MMAD, and GSD (5-fold cross-validation).

ML Algorithms	RF			XGB			SVM			MLP		
	Train	Test	CV	Train	Test	CV	Train	Test	CV	Train	Test	CV
FPD_R^2^	0.974	0.961	0.937	0.997	0.964	0.928	0.980	0.965	0.945	0.975	0.967	0.943
FPD_MAE	111.594	128.873	185.426	39.643	133.893	189.717	104.331	129.216	169.274	108.597	121.438	160.303
FPD_RMSE	149.713	171.962	229.030	54.636	163.984	245.182	131.444	161.969	210.994	146.954	158.204	213.571
FPF_R^2^	0.988	0.966	0.963	0.999	0.991	0.980	0.988	0.950	0.973	0.989	0.968	0.969
FPF_MAE	1.993	2.692	3.876	0.624	1.547	2.612	2.243	3.360	3.222	1.871	2.620	2.755
FPF_RMSE	2.743	3.898	4.557	0.798	1.997	3.492	2.770	4.678	4.064	2.626	3.737	4.124
MMAD_R^2^	0.984	0.966	0.952	0.996	0.982	0.922	0.978	0.939	0.934	0.982	0.976	0.963
MMAD_MAE	0.083	0.107	0.171	0.040	0.105	0.196	0.125	0.184	0.193	0.112	0.117	0.156
MMAD_RMSE	0.164	0.212	0.268	0.080	0.154	0.345	0.192	0.284	0.306	0.174	0.178	0.217
GSD_R^2^	0.964	0.898	0.910	0.997	0.894	0.893	0.967	0.881	0.904	0.961	0.903	0.890
GSD_MAE	0.022	0.041	0.034	0.007	0.038	0.037	0.020	0.041	0.036	0.022	0.040	0.035
GSD_RMSE	0.029	0.048	0.042	0.009	0.049	0.048	0.028	0.052	0.046	0.030	0.047	0.049

**Table 4:** The selected optimal formulation of rifampicin and pyrazinamide (RIF: rifampin, PYR: pyrazinamide, LLA: L-Lysine Acetate, LL: L-leucin).

Formulation	Atomizer (L/h)	Ratio of Drug to Amino Acid	FPD (µg)	Predicted FPD	FPF (%)	Predicted FPF	MMAD (µm)	Predicted MMAD	GSD	Predicted GSD
RIF-LLA	850	1:1	3127.32	3159.09	73.08	72.77	2.63	2.65	1.65	1.65
PYR-LL	850	1:1	2521.71	2471.32	86.31	85.40	1.88	1.89	1.84	1.84

## Data Availability

Data are available on reasonable request from the corresponding authors.

## Supplementary Materials

The following supporting information can be downloaded at: https://www.mdpi.com/article/10.3390/pharmaceutics18020191/s1, Table S1: HPLC conditions; Table S2: Aerodynamic assessment results of 36 RIF formulations; Table S3: Aerodynamic assessment results of 36 PYR formulations; Figure S1: Normalized heatmap of feature importance in predictive models.
