# Supplementary material for: Machine Learning-Guided Development of Anti-Tuberculosis Dry Powder for Inhalation Prepared by Co-Spray Drying
Source: Pharmaceutics. 2026 Feb 1;18(2):191. doi: 10.3390/pharmaceutics18020191 (PMC12943867; doi:10.3390/pharmaceutics18020191)
Supplement: Supplementary file 1 [file pharmaceutics-18-00191-s001.zip › pharmaceutics-4099849-supplementary.pdf]

Supplementary Materials: Machine Learning-Guided Development of Anti-Tuberculosis Dry Powder for Inhalation Prepared by Co-Spray Drying

Table S1. HPLC conditions.

Formulation	Rifampin DPIs	Pyrazinamide DPIs
Equipment	Shimadzu LC-20T or Agilent 1260	Shimadzu LC-20T or Thermo U3000
Column	Kromasil C_8 column (4.6 × 125 mm, 5 µm; Sweden)	Thermo BDS HYPRERSIL C_{18} (4.6 × 200 mm, 5 µm; USA)
Mobile phase A	Methanol:Acetonitrile (50:50, v/v)	pH 3.0 acetic acid aqueous solution
Mobile phase B	0.035 mol/L potassium dihydrogen phosphate buffer solution: 0.5mol/L citric acid aqueous solution (90:10, v/v)	Methanol
Ratio of phases	Mobile phase A:B (61:39, v/v)	Mobile phase A:B (90:10, v/v)
Flow rate	1.2 ml/min	1.2 ml/min
Sample volume	10 µl	20 µl
Wavelength	254 nm	268 nm
Linearity range	0.0811-162.2400 µg/ml; Y = 18856.0317X + 9536.1456; R^2=1.0000	0.0302-120.8800 µg/ml; Y = 0.9971X + 0.4517; R^2=0.9998

Table S2. Aerodynamic assessment results of 36 RIF formulations (*n* = 3, RIF: rifampin, LA: L-arginine, LLA: L-Lysine Acetate, LL: L-leucine, AA: amino acid).

Formulation	Atomizer(L/h)	Ratio of rug-AA	FPD (μg)	FPF (%)	MMAD (μm)	GSD	EF (%)	ED (μg)
RIF-LA	850	2:1	2214.36±19.94	50.70±1.02	3.17±0.00	1.70±0.03	85.70±2.49	4368.44±98.10
		1:1	2473.71±10.34	52.58±0.42	2.90±0.06	1.74±0.02	88.90±4.13	4704.45±19.41
		1:2	1878.80±112.93	55.65±1.66	2.71±0.02	1.76±0.03	88.47±0.19	3374.14±105.56
	800	2:1	2300.68±53.46	47.74±1.63	2.95±0.06	1.77±0.00	89.31±1.16	4821.14±141.34
		1:1	2246.82±126.28	50.49±1.47	3.18±0.13	1.69±0.02	83.48±2.95	4447.61±123.09
		1:2	2442.93±39.91	52.87±0.59	2.91±0.08	1.73±0.02	87.55±3.17	4620.82±24.08
	750	2:1	2064.49±76.01	50.34±2.50	3.19±0.03	1.66±0.02	89.16±0.87	4102.72±52.42
		1:1	2255.17±67.61	50.40±0.78	3.17±0.07	1.69±0.01	83.54±1.62	4473.57±70.45
		1:2	2101.54±68.22	52.40±1.23	3.19±0.05	1.65±0.02	89.01±1.77	4010.31±44.74
	700	2:1	2015.00±144.92	40.77±3.70	3.12±0.15	1.79±0.02	90.18±1.57	4951.81±249.42
		1:1	1900.43±17.86	46.44±1.30	3.22±0.02	1.69±0.02	89.15±0.78	4093.33±83.32
		1:2	2028.76±30.58	47.19±0.50	3.21±0.06	1.72±0.02	82.69±1.62	4298.86±27.28
RIF-LLA	850	2:1	2791.30±17.42	70.60±1.03	2.64±0.01	1.66±0.00	81.76±1.83	3954.09±52.30
		1:1	3127.32±50.35	73.08±0.60	2.63±0.01	1.65±0.00	83.03±1.75	4280.12±104.48
		1:2	3121.15±98.32	73.08±1.21	2.63±0.02	1.65±0.00	83.07±3.44	4273.42±204.63
	800	2:1	2980.39±107.69	71.30±2.80	2.60±0.01	1.68±0.01	83.87±1.07	4180.68±12.94
		1:1	3087.69±40.09	73.27±0.42	2.63±0.01	1.65±0.00	83.35±1.04	4214.53±78.46
		1:2	3085.00±121.39	73.37±2.17	2.59±0.01	1.68±0.01	82.62±0.52	4203.99±58.15
	750	2:1	3093.58±103.12	73.25±1.21	2.63±0.02	1.65±0.00	83.06±3.46	4225.64±209.35
		1:1	3031.87±31.57	71.67±0.75	2.60±0.00	1.69±0.00	83.59±0.32	4230.33±5.99
		1:2	2828.7±48.03	68.26±1.60	2.61±0.01	1.69±0.01	83.97±0.79	4144.79±89.95
	700	2:1	2530.53±84.10	66.92±2.11	2.63±0.01	1.70±0.02	82.34±0.80	3781.42±18.92
		1:1	2877.32±63.04	70.43±0.66	2.64±0.01	1.66±0.00	82.96±1.87	4085.95±116.39
		1:2	3000.80±183.17	71.51±4.47	2.60±0.02	1.68±0.01	83.75±1.53	4196.86±9.10

Formulation	Atomizer(L/h)	Ratio of rug-AA	FPD (μg)	FPF (%)	MMAD (μm)	GSD	EF (%)	ED (μg)
RIF-LL	850	2:1	2583.03±100.73	57.91±4.18	3.02±0.04	1.71±0.01	86.91±1.11	4467.07±143.00
		1:1	2664.77±14.47	63.45±0.84	2.98±0.04	1.61±0.04	84.94±1.00	4200.40±34.58
		1:2	2917.04±34.70	66.40±1.51	2.97±0.07	1.61±0.07	85.50±2.21	4394.24±52.97
	800	2:1	2591.25±196.72	59.94±8.48	3.02±0.09	1.71±0.02	86.71±2.42	4453.29±225.11
		1:1	2653.91±34.83	63.68±1.36	2.97±0.02	1.60±0.04	85.09±1.05	4168.14±36.11
		1:2	2926.68±18.81	66.97±0.52	2.98±0.04	1.59±0.05	85.22±1.29	4370.24±23.47
	750	2:1	2885.74±40.45	66.41±1.27	2.96±0.04	1.60±0.05	85.74±1.55	4345.65±30.25
		1:1	2681.43±36.11	62.62±1.15	2.98±0.06	1.72±0.03	85.37±0.94	4282.39±21.32
		1:2	2899.36±35.74	66.50±1.60	2.97±0.07	1.60±0.07	85.60±2.24	4359.80±51.07
	700	2:1	2223.22±77.96	51.66±2.24	3.08±0.01	1.73±0.01	87.35±0.52	4305.10±62.30
		1:1	2441.84±96.91	54.34±4.32	3.06±0.05	1.72±0.01	87.72±1.60	4503.31±183.26
		1:2	2187.68±112.43	51.27±2.39	3.05±0.05	1.74±0.01	86.84±0.53	4266.28±35.25

Table S3. Aerodynamic assessment results of 36 PYR formulations (n = 3, PYR: pyrazinamide, LA: L-arginine, LLA: L-Lysine Acetate, LL: L-leucine, AA: amino acid).

Formulation	Atomizer(L/h)	Ratio of rug-AA	FPD (μg)	FPF (%)	MMAD (μm)	GSD	EF (%)	ED (μg)
PYR-LA	850	2:1	208.16±11.98	6.41±0.33	5.98±0.12	2.05±0.03	96.76±0.06	3246.98±24.98
		1:1	220.10±7.89	7.43±0.28	5.61±0.08	2.00±0.01	96.74±0.01	2961.02±23.72
		1:2	245.86±19.83	7.45±0.48	5.61±0.14	2.02±0.02	96.73±0.02	3298.88±54.19
	800	2:1	223.13±6.78	7.11±0.18	5.68±0.05	2.01±0.03	96.75±0.01	3139.54±18.57
		1:1	219.07±6.09	6.88±0.18	5.76±0.06	2.02±0.02	96.73±0.01	3183.67±7.29
		1:2	248.61±10.56	7.62±0.29	5.56±0.06	2.01±0.03	96.74±0.01	3261.19±19.67
	750	2:1	171.43±2.77	5.89±0.16	6.16±0.02	2.04±0.02	96.78±0.15	2910.90±30.96
		1:1	183.73±7.21	6.36±0.21	4.97±1.77	2.08±0.02	96.70±0.01	2889.57±30.68
		1:2	185.72±7.04	6.09±0.18	6.04±0.09	2.04±0.03	96.88±0.15	3050.33±30.60
	700	2:1	150.88±4.40	4.87±0.18	6.45±0.02	2.13±0.01	96.62±0.02	3100.03±28.33
		1:1	177.93±6.60	5.81±0.19	6.21±0.08	2.08±0.02	96.67±0.00	3061.97±23.70
		1:2	160.74±3.76	5.43±0.08	6.39±0.03	2.11±0.02	96.66±0.02	2959.77±30.71
PYR-LLA	850	2:1	1473.29±41.84	53.81±0.64	3.41±0.01	1.65±0.01	94.86±0.26	2737.76±44.97
		1:1	1613.91±79.62	55.68±1.25	3.39±0.04	1.63±0.01	95.06±0.52	2897.53±77.98
		1:2	1632.12±50.78	55.98±0.79	3.38±0.02	1.63±0.01	95.21±0.34	2915.26±49.74
	800	2:1	1651.72±107.73	52.47±1.93	3.49±0.07	1.57±0.02	80.13±0.06	3145.91±91.49
		1:1	1530.43±53.20	50.67±1.12	3.51±0.02	1.58±0.01	79.56±0.06	3019.97±39.38
		1:2	1618.79±79.62	55.75±1.26	3.39±0.04	1.63±0.01	95.11±0.53	2902.40±77.46
	750	2:1	1446.85±23.39	51.89±0.44	3.43±0.02	1.65±0.01	94.90±0.22	2788.03±21.22
		1:1	1467.59±10.60	49.74±0.19	3.50±0.01	1.60±0.00	79.27±0.05	2950.72±11.41
		1:2	1597.21±48.52	51.88±0.79	3.46±0.03	1.58±0.01	80.00±0.09	3077.97±48.35
	700	2:1	1506.93±55.48	50.68±0.87	3.49±0.05	1.59±0.02	79.36±0.22	2972.89±58.07
		1:1	1588.10±105.07	51.51±1.90	3.46±0.08	1.58±0.02	80.09±0.11	3080.87±88.33
		1:2	1398.84±47.96	51.16±0.81	3.43±0.00	1.66±0.02	94.75±0.35	2733.51±51.14

Formulation	Atomizer(L/h)	Ratio of rug-AA	FPD (μg)	FPF (%)	MMAD (μm)	GSD	EF (%)	ED (μg)
PYR-LL	850	2:1	2343.48±24.92	84.58±0.23	1.89±0.02	1.84±0.01	90.89±0.24	2770.63±22.32
		1:1	2521.71±94.65	86.31±1.49	1.88±0.00	1.84±0.01	90.82±0.37	2838.30±104.29
		1:2	2514.49±142.71	87.62±0.19	1.88±0.01	1.83±0.00	91.11±0.13	2872.94±151.92
	800	2:1	2105.05±184.20	82.31±1.40	1.89±0.01	1.86±0.01	89.81±0.35	2556.07±189.10
		1:1	2116.72±159.29	79.91±0.23	1.89±0.02	1.85±0.03	90.02±0.37	2649.23±203.39
		1:2	2674.31±284.72	87.74±0.03	1.88±0.00	1.83±0.00	91.13±0.08	2858.04±105.94
	750	2:1	2053.68±87.55	83.04±0.09	1.89±0.03	1.84±0.01	89.93±0.05	2473.20±108.15
		1:1	2236.94±90.64	84.56±0.42	1.88±0.00	1.85±0.00	90.43±0.14	2644.94±94.23
		1:2	2053.90±105.43	83.07±0.26	1.90±0.01	1.85±0.02	89.69±0.19	2472.31±119.64
	700	2:1	1777.31±41.25	77.71±0.26	1.89±0.01	1.86±0.03	88.75±0.35	2287.26±54.95
		1:1	1803.51±14.92	74.20±0.26	1.90±0.03	1.86±0.01	89.40±0.21	2430.46±19.17
		1:2	1936.22±103.28	79.05±1.03	1.89±0.02	1.87±0.01	89.47±0.21	2448.54±106.18

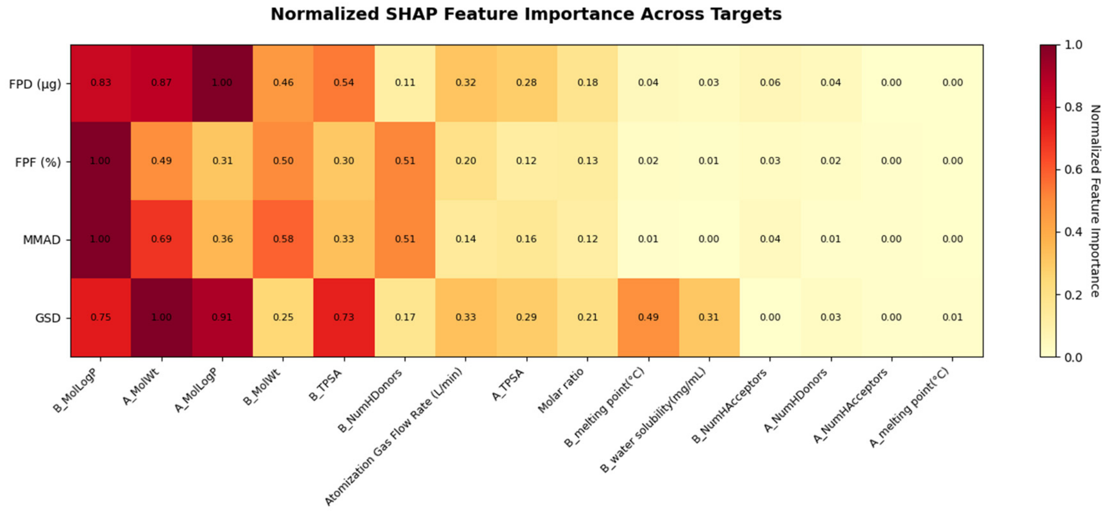

Figure S1. Normalized heatmap of feature importance in predictive models.
